# Editorial: Human Papillomaviruses and Polyomaviruses in Skin Cancer

**DOI:** 10.3389/fmicb.2018.02778

**Published:** 2018-11-15

**Authors:** Herbert Johannes Pfister, Marisa Gariglio, Sigrun Smola

**Affiliations:** ^1^Institute of Virology, Universität zu Köln, Cologne, Germany; ^2^Scuola di Medicina, Università degli Studi del Piemonte Orientale, Novara, Italy; ^3^Institute of Virology, Saarland University, Saarbrücken, Germany

**Keywords:** papillomavirus, polyomavirus, nonmelanoma skin cancer, Merkel cell carcinoma, cutaneous squamous cell carcinoma

Nonmelanoma skin cancer (NMSC), more recently defined as keratinocyte carcinoma (KC), is the most frequent malignant tumor among Caucasians. NMSC comprises two major types of skin cancer: basal cell carcinoma (BCC) and cutaneous squamous cell carcinoma (cSCC), accounting for 80 and 20% of all NMSC patients, respectively. Intriguingly, the incidence of cSCC in immunosuppressed organ transplant recipients is 60–250 times higher than that observed in the general population, which is highly suggestive of viral etiology. An etiologic role of human papillomaviruses (HPV) of the genus beta (betaPV) has long been proposed due to the well-documented carcinogenicity of these cutaneous viruses in patients suffering from epidermodysplasia verruciformis (EV) (Quint et al., 2015; Hufbauer and Akgül, 2017). In addition, two epidemiologic prospective studies have recently shown the presence of anti-betaPV antibodies around the time of transplantation to be predictive of keratinocyte carcinoma development in organ transplant recipients (Genders et al., 2015), and both betaPV type diversity and viral DNA load in plucked eyebrow hair to be positively associated with an almost doubling of the risk of developing cSCC (Bouwes Bavinck et al., 2018).

Besides epidemiological evidence, mechanistic studies have reported multiple potentially oncogenic properties of betaPV proteins in keratinocyte cultures as well as transgenic mice, thus providing *in vitro* and *in vivo* functional data corroborating betaPV carcinogenicity. The proposed mechanisms indicate that betaPV play a causative role in tumor initiation and progression, but are not necessary for tumor maintenance, the so-called “hit and run” theory. This type of oncogenic activity of betaPV in cSCC differs profoundly from that of alphaPV. It is generally accepted that deregulated expression of the oncoproteins E6 and E7 of the high-risk mucosotropic alphaPVs such as HPV16 and 18 underlies neoplasia and eventual progression to anogenital or oropharyngeal cancers. The E6 and E7 oncoprotein expression is usually maintained in the full-blown cancers, indicating their necessary role in tumor maintenance. The paradigm shift between alphaPV and betaPV in terms of their role in tumor maintenance has been very important to appreciate the potential role of betaPV in skin cancer.

Two members of the human polyomavirus family (HPyV) have been associated with either malignant or benign skin tumors, respectively: the Merkel cell polyomavirus (MCPyV) has been identified and cloned from Merkel cell carcinoma (MCC) and has been reported to be monoclonally integrated in up to 80% of MCC cases, while the *Trichodysplasia spinulosa* Polyomavirus (TSPyV) is generally regarded as the causative agent of the homonymous, very rare but rather disfiguring, benign proliferative skin disease. To date, there are three more human polyomaviruses with skin-tropism but unknown pathogenicity, so far.

In summary, the Research Topic deals with widely distributed cutaneous papillomaviruses and polyomaviruses, which are able to establish life-long persistent infections. Although they are frequently clinically inapparent in the general population, they can cause skin cancer in immunocompromised hosts or in the aging skin undergoing immunosenescence, thereby leading to major medical problems.

Given the well-established carcinogenicity of betaPV in EV patients, the Research Topic opens with a mini review by De Jong et al. highlighting inborn errors of immunity as the genetic basis for the spectacular susceptibility of EV patients to widespread betaPV infection, resulting in cSCC formation in 30–60% of infected patients.

Among the molecular mechanisms contributing to betaPV-induced oncogenesis, attention first focused on the inhibition of cellular DNA damage repair pathways and apoptosis by the viral E6 protein, leading to genomic instability and accumulation of harmful mutations. The betaPV E6 proteins also interfere with Notch and C/EBPalpha/mir-203 signaling in keratinocytes, thereby with keratinocyte differentiation, and possibly contributing to continued proliferation of the infected cells. These activities are comprehensively reviewed and discussed by Wendel and Wallace and Meyers et al, respectively. Meyers et al. point out that the genus gamma HPV197, which was frequently detected in cSCC by next-generation sequencing, shares many cellular targets of its E6 and E7 proteins with E6/E7 of beta PV.

The Research Topic also boasts three original research contributions reporting new potentially oncogenic functions of betaPV. Marx et al. show that HPV8-E6 can interfere with cellular syntenin-2 gene regulation and its role in keratinocyte differentiation. Taute et al. report that HPV8-E6 can also stimulate epidermal growth factor receptor (EGFR) signaling in response to UV irradiation, and that this effect is required for papilloma formation in UV-irradiated mice. Lastly, Podgórska et al. demonstrate that HPV8-E2 together with C/EBPbeta strongly enhances S100A8/A9 protein expression, which in turn drives tumor promoting inflammation as observed in lesional skin from EV patients.

The following “Perspective” section by Olivero et al. identifies which betaPV target cells are critical for viral carcinogenesis. The authors show that when betaPV persistently infects adult tissue stem cells, it induces their proliferation and displacement beyond the stem cell niche as a first step toward field cancerization. Once the HPV-infected cells have acquired a number of transforming mutations, the viral DNA is not any longer required for malignant conversion and may get lost. This scenario has been revealed before by original research (Lanfredini et al., 2017). In good agreement, Borgogna et al. show here that one betaPV-positive hyperplasia from a kidney transplant recipient progressed to an HPV-negative, metastasizing SCC after xenografting in nude mice.

The thoughtful and critical review by Hasche et al. summarizes current data supporting the viral etiology of NMSC, and it also reports current work and perspectives on vaccination against cutaneous HPVs. In this regard, it is pleasantly rewarding for the scientific community to come to the realization that the viral etiology of NMSC is finally being accepted by public health institutions to a degree, which will allow the clinical development of vaccines. It goes without saying that a reduced incidence of NMSC in immunocompromised individuals after vaccination would be the litmus test of its viral etiology.

In striking contrast to what happens in cSCC in terms of betaPV infection, continued expression of the MCPyV T antigen is essential for cell proliferation in most virus-positive MCC cell lines. In this regard, Velásquez et al. here describe a newly established MCPyV-positive cell line requiring T antigen expression to proliferate. MCPyV and TSPyV encode in addition to (Large) T antigen, Middle (MT), and alternative (ALT) T antigens. Van der Meijden and Feltkamp here speculate on their roles and relevance to cancer.

The wide distribution of cutaneous papillomaviruses and polyomaviruses has raised, and still raises, a number of unsolved issues in epidemiologic case-control studies apart from MCC, where the virus is always found integrated into the cellular genome and mutated. These issues become rather obvious in the last two contributions of the Research Topic. Although Hillen et al. detect MCPyV in seborrheic keratosis, the authors conclude that this observation mainly reflects its widespread occurrence in the skin rather than representing a cause-effect link between seborrheic keratosis and the virus. Finally, Purdie et al. report a virologically broad investigation of HPV and five skin-tropic human polyomaviruses in BRAF-inhibitor (BRAFi)-induced cSCC. This drug, which has been in clinical use since 2011 for the treatment of metastatic melanoma, can induce, alongside other adverse effects, cSCC formation with a median lag time of only 8–12 weeks. Interestingly, the clinicopathological characteristics of these cSCCs lead the authors to speculate on a possible involvement of oncogenic viruses. Indeed, betaPV, MCPyV, and HPyV7 were detected in 66, 73, and 55% of cSCCs, respectively. Overall, the three viruses were co-detected in 55% of the tumors. However, they conclude that a potential role of HPV and/or HPyV in BRAFi-induced cSCC still remains uncertain and warrants further investigation.

To sum up, this volume comprises a large body of evidence attesting the viral etiology of NMSC. Nonetheless, there are still many unanswered questions awaiting further investigation. Based on the evidence hereby presented, we believe that future research efforts should take into account the following main points emerging from this volume:

1. The detection of HPV197 DNA in human skin cancer, together with the observation that its E6/E7 proteins interact with human tumor suppressor proteins, strongly suggests that this virus and possibly other gammaPVs may play a more important role in skin carcinogenesis than ever thought before;
2. In light of the proposed “hit and run” mechanism of betaPV-driven skin carcinogenesis, further research focusing on the early steps of betaPV infection is necessary to identify new diagnostic markers as well as potential targets for anticancer therapy;
3. Given the medical relevance of BRAFi-induced NMSC development, further research into the role of the HPV and HPyV identified so far and possibly other oncogenic viruses appear justified. Possible synergistic interactions between the frequently codetected HPV and HPyV would be of great interest in medical and general virology.
4. Finally, the evidence presented in this volume indicates that highest priority should be given to the implementation of a prophylactic and/or therapeutic vaccination program against cutaneous HPV to prevent NMSC in immunosuppressed allograft recipients. Consequently, basic research on viral life cycle and oncogenic functions, research on vaccine formulations, and challenge experiments in vaccinated animal models should all be given the needed resources and recognition. We strongly believe that the time is now ripe for this essential translational step in HPV-related skin cancer research.

## Author contributions

All authors listed have made a substantial, direct and intellectual contribution to the work, and approved it for publication.

### Conflict of interest statement

The authors declare that the research was conducted in the absence of any commercial or financial relationships that could be construed as a potential conflict of interest.
